# Differential effects of cigarette smoke on oxidative stress and proinflammatory cytokine release in primary human airway epithelial cells and in a variety of transformed alveolar epithelial cells

**DOI:** 10.1186/1465-9921-7-132

**Published:** 2006-10-24

**Authors:** Aruna Kode, Se-Ran Yang, Irfan Rahman

**Affiliations:** 1 Department of Environmental Medicine, Lung Biology and Disease Program, University of Rochester Medical Center, Rochester, NY, USA

## Abstract

**Background:**

Cigarette smoke mediated oxidative stress and inflammatory events in the airway and alveolar epithelium are important processes in the pathogenesis of smoking related pulmonary diseases. Previously, individual cell lines were used to assess the oxidative and proinflammatory effects of cigarette smoke with confounding results. In this study, a panel of human and rodent transformed epithelial cell lines were used to determine the effects of cigarette smoke extract (CSE) on oxidative stress markers, cell toxicity and proinflammatory cytokine release and compared the effects with that of primary human small airway epithelial cells (SAEC).

**Methods:**

Primary human SAEC, transformed human (A549, H1299, H441), and rodent (murine MLE-15, rat L2) alveolar epithelial cells were treated with different concentrations of CSE (0.2–10%) ranging from 20 min to 24 hr. Cytotoxicity was assessed by lactate dehydrogenase release assay, trypan blue exclusion method and double staining with acridine orange and ethidium bromide. Glutathione concentration was measured by enzymatic recycling assay and 4-hydroxy-2-nonenal levels by using lipid peroxidation assay kit. The levels of proinflammatory cytokines (e.g. IL-8 and IL-6) were measured by ELISA. Nuclear translocation of the transcription factor, NF-κB was assessed by immunocytochemistry and immunoblotting.

**Results:**

Cigarette smoke extract dose-dependently depleted glutathione concentration, increased 4-hydroxy-2-nonenal (4-HNE) levels, and caused necrosis in the transformed cell lines as well as in SAEC. None of the transformed cell lines showed any significant release of cytokines in response to CSE. CSE, however, induced IL-8 and IL-6 release in primary cell lines in a dose-dependent manner, which was associated with the nuclear translocation of NF-κB in SAEC.

**Conclusion:**

This study suggests that primary, but not transformed, lung epithelial cells are an appropriate model to study the inflammatory mechanisms in response to cigarette smoke.

## Background

Cigarette smoke, a complex admixture of more than 4700 chemical compounds and oxidants [1], is an important etiological factor in the development of chronic obstructive pulmonary disease (COPD). It contains 10^14^–10^16 ^free radicals/puff, which include reactive aldehydes, quinones and benzo(a)pyrene [2]. Many of these are relatively long lived, such as tar-semiquinone, which can also generate hydroxyl radicals (•OH) and hydrogen peroxide (H_2_O_2_) by Fenton reaction in presence of free iron. These agents induce an oxidative burden by disturbing the oxidant:antioxidant balance and could lead to cellular damage in the lungs. Oxidative stress caused by cigarette smoking can result in destruction of the alveolar wall, leading to airway enlargement. Moreover, increased oxidative stress can trigger proinflammatory cytokines, which are increased in the lungs of smokers and patients with COPD [3,4].

The airway/airspace epithelium is the primary target for any inhaled environmental agents and plays a critical role in the release of pro-inflammatory mediators. It is also involved in the progression of tissue injury during inflammatory conditions, implicating the role of airway/airspace epithelium in the pathogenesis of inflammatory airway diseases such as COPD. Previous *in vivo *findings have supported the above, wherein; cigarette smoke was shown to induce proinflammatory cytokine release in smokers and in rodent lungs [5,6]. However, the precise molecular mechanism as how cigarette smoke generates signals for proinflammatory cytokine release, particularly in airway or alveolar epithelium is not yet clearly understood.

Earlier, we have demonstrated the ability of cigarette smoke extract (CSE) to induce oxidative stress in transformed human alveolar epithelial cells (A549), which could not be correlated to the release of any proinflammatory cytokines [7,9]. A549 is the most widely used cell line and is well criticized in the literature [10]. In this study, we investigated whether cigarette smoke can trigger proinflammatory cytokine release in any other alveolar epithelial cell lines derived from either human or rodents. To test our hypothesis, we used a panel of human and rodent alveolar epithelial cell lines, such as human lung cancer cells (H1299), human lung epithelial cells (H441), murine type II epithelial cells (MLE-15), and rat lung epithelial cells (L2) in addition to human adenocarcinoma cells (A549). Another aim of this study was to develop an *in vitro *cell culture model for understanding the mechanisms of proinflammatory effects of cigarette smoke exposure. To this extent, we studied the effect of CSE on oxidative stress (reduced glutathione and 4-hydroxy-2-nonenal), cell toxicity (lactate dehydrogenase release, apoptosis and necrosis) and proinflammatory cytokine release (IL-6 and IL-8) in various transformed epithelial cell lines and in primary human small airway epithelial cells.

## Materials and methods

All biochemicals were of analytical grade and purchased from Sigma Chemical Co (St. Louis, MO) unless otherwise stated.

### Materials

Penicillin, streptomycin and culture media (DMEM, RPMI 1640, F12K) were procured from Life technologies (Gaithersburg, MD, USA). Fetal bovine serum (FBS) was obtained from HyClone Laboratories (Logan, UT, USA). Rabbit polyclonal anti NF-κB Rel/p65 antibody (sc-372) was purchased from Santa Cruz Biotechnology Inc., (Santa Cruz, CA, USA).

### Cell culture

Five different alveolar epithelial type II cell lines were used for this study along with the primary human small airway epithelial cells (SAEC). The sources of various cell lines were as follows: the human adenocarcinoma epithelial cells (A549) derived from lungs of adenocarcinoma patient, human lung epithelial cells from papillary adenocarcinoma patient (H441), human lung cancer cells from cancer patient (H1299), and rat lung epithelial cells (L2) were obtained from American Type Cell Collection (ATCC), Manassas, VA, USA. Murine type II epithelial cells (MLE-15) were derived from immortalized lung tumors of transgenic mice containing the simian virus 40 large T antigen under the transcriptional control of the regulatory sequences derived from the human surfactant protein (SP)-C promoter region [11,12]. Cells were grown in culture media (A549 and H1299: Dulbecco's modified Eagle medium, H441: RPMI 1640 medium, MLE-15: DMEM/F12K medium and L2: F12K medium) supplemented with 10% FBS, 2 mM L-glutamine, 100 IU/ml penicillin, 100 μg/ml streptomycin at 37°C in a humidified atmosphere containing 5% CO_2_.

SAEC derived from a single healthy non-smoker, and the basal media (SAGM) including all the growth supplements were purchased from Clonetics (San Diego, CA, USA). Cells were cultured according to the supplier's instructions. Passage number was kept to less than seven passages from original stocks. SAEC were maintained in SAGM supplemented with 52 μg/ml bovine pituitary extract, 0.5 ng/ml human recombinant epidermal growth factor (EGF), 0.5 μg/ml epinephrine, 10 μg/ml transferrin, 5 μg/ml insulin, 0.1 ng/ml retinoic acid (RA), 6.5 ng/ml triiodothyronine, 50 μg/ml Gentamicin/Amphotericin-B (GA-1000), and 50 μg/ml fatty acid-free bovine serum albumin (BSA). Polymyxin B sulfate, an endotoxin binding agent (10 μg/ml), was also included in the media to prevent lipopolysaccharide contamination [13].

### Preparation of aqueous cigarette smoke extract

Research grade cigarettes (1R3F) were obtained from the Kentucky Tobacco Research and Development Center at the University of Kentucky, Lexington, KY, USA. The composition of 1R3F research grade cigarettes was: total particulate matter: 17.1 mg/cigarette, tar: 15 mg/cigarette and nicotine: 1.16 mg/cigarette. Cigarette smoke extract (10%) was prepared by bubbling smoke from one cigarette into 10 ml of culture media supplemented with 1% FBS at a rate of one cigarette/minute as described previously [9,13], using a modification of the method described earlier by Carp and Janoff [14]. The pH of the CSE was adjusted to 7.4, and was sterile filtered through a 0.45 μm filter (25 mm Acrodisc; Pall Corporation, Ann Arbor, MI). Cigarette smoke extract preparation was standardized by measuring the absorbance (OD 0.74 ± 0.05) at a wavelength of 320 nm. The pattern of absorbance (spectrogram) observed at λ_320 _showed a very little variation between different preparations of CSE. Cigarette smoke extract was freshly prepared for each experiment and diluted with culture media supplemented with 1% FBS immediately before use. Control medium was prepared by bubbling air through 10 ml of culture media supplemented with 1% FBS, and the pH was adjusted to 7.4, and sterile filtered as described above.

### Cell treatments

Epithelial cells (H1299, A549, H441, MLE-15 and L2) were seeded at a density of 1.5 million cells in 6-well plates containing culture media supplemented with 10% FBS in a final volume of 2 ml. The cells were grown to approximately 80–90% confluency, then changed to 1% FBS during the treatment. All treatments were performed in duplicate. The cells were treated with CSE (1.0–10%) for 24 hr at 37°C in a humidified atmosphere containing 5% CO_2_. 10 ng/ml tumor necrosis factor-α (TNF-α), was used as a positive control in selected experiments [15]. After 24 hr treatment, cell supernatants were collected for LDH release and proinflammatory cytokines (interleukin-8 and interleukin-6) assays. Cell lysates were prepared for GSH and 4-HNE assays. Similarly, the epithelial cells were grown in 8-well chamber slides and treated with CSE (1.0–10%) for 24 hr and stained with a solution comprising of acridine orange and ethidium bromide dyes for apoptotic and necrotic studies.

Human SAEC were seeded in 12-well plates containing SAGM. After reaching 80% confluency, the cells were treated with either TNF-α (10 ng/ml) or CSE (0.2–1.0%); as higher doses (>1.0%) were cytotoxic to the cells. After the incubation period, the culture media was collected for LDH release and proinflammatory cytokines (IL-8 and IL-6) assay. Cell lysates were prepared for GSH, 4-HNE assays and western blotting for p65 protein. Primary cells were also grown in 8-well chamber slides, treated as described above, and were fixed with 4% paraformaldehyde for the detection of NF-κB nuclear translocation.

### Cytotoxicity assay

Cell toxicity was assessed by three separate methods: LDH release assay, trypan blue exclusion method and double staining with acridine orange and ethidium bromide.

### Lactate dehydrogenase assay

LDH release, an indicator of membrane integrity and viability of alveolar epithelial cells, was measured in various treated samples, and compared with control (untreated) cultures using a commercially available LDH cytotoxicity assay kit (Roche Diagnostics, Indianapolis, USA). Following treatments, the culture medium was collected and centrifuged at 5000 rpm for 5 min prior to analysis. Assay was performed according to the manufacturer's instructions. LDH release was quantified by measuring the absorbance at 490 nm using a microplate reader (Bio-Rad, Hercules, CA, USA). A 100% lysis control was prepared by adding 1% Triton-X-100 to control cell pellet to release all LDH. The absorbance value obtained was used for calculating percentage cytotoxicity.

### Trypan blue exclusion assay

After 24 hr incubation, the culture medium was removed and replaced by 0.1% trypan blue solution in Ca^2+^/Mg^2+^-free phosphate buffered saline (PBS) for 3 min at room temperature. The cells stained blue were considered non-viable cells, whereas the cells that excluded the stain were considered viable.

### Assay of apoptosis and necrosis

Morphological evidence of apoptosis and necrosis was obtained by means of acridine orange and ethidium bromide staining as described previously [16,17]. In brief, after treatment, cells in 8-well chamber slides were stained with acridine orange (4 μg/ml) and ethidium bromide (4 μg/ml). Cells were examined by fluorescence microscopy (Olympus BX51 microscope, New Hyde Park, NY, USA), and photographed using a SPOT camera with SPOT RT software (Olympus). Acridine orange permeates throughout the cells and renders the nuclei green. Ethidium bromide is taken up by the cells only when cytoplasmic membrane integrity is lost, and stains the nuclei red. Viable (normal, green nuclei), early apoptotic (condensed, green nuclei), late apoptotic (condensed, red nuclei) and necrotic (normal, red nuclei) cells were quantified by counting a minimum of 100 cells in total in three independent experiments.

### Measurement of intracellular 4-hydroxy-2-nonenal levels

4-HNE levels were measured in cell lysates by using lipid peroxidation assay kit (Calbiochem, San Diego, CA, USA). After the treatment period, cells were rinsed twice with ice-cold PBS and scraped off using cell scrapers (Sarsdet Inc. Newton, NC, USA). The pellet was resuspended in 200 μl of 20 mM Tris-HCl, pH 7.4, containing 5 mM butylated hydroxytoluene, and kept frozen at -70°C until assayed. To each sample, 650 μl of N-methyl-2-phenylindole and 150 μl of 15.4 M methanesulfonic acid were added. The reaction mixture was vortexed and incubated at 45°C for 60 min. After centrifugation at 15000 *g *for 10 min, the absorbance of the supernatant was determined at 586 nm. The levels of 4-HNE were determined from standard calibration curve constructed using 4-HNE diethylacetal in methanesulfonic acid. The values were expressed as μmol 4-HNE/mg protein.

### Measurement of intracellular glutathione levels

Intracellular GSH levels in the cell extracts were measured by the 5,5'-dithiobis-2-nitrobenzoic acid DTNB-GSSG reductase recycling method described by Tietze [18] with slight modifications [8,19,20]. In brief, the cells were rinsed twice with ice-cold PBS, scraped off from the 6 well plate, suspended into 500 μl of ice-cold extraction buffer (0.1% Triton X-100 and 0.6% sulfosalicylic acid prepared in 0.1 M phosphate buffer with 5 mM EDTA, pH 7.5). The cells were vortexed for 20 seconds, followed by sonication (30 seconds) and centrifugation (2500 rpm for 5 min at 4°C). Twenty microlitres of the supernatant was added to 120 μl of 0.1 M phosphate buffer, 5 mM EDTA, pH 7.5, containing 100 μl of 5 mM DTNB and 0.5 units of glutathione reductase. Finally 60 μl of 2.4 mM NADPH was added and the rate of change in absorbance was measured for 1 min at 410 nm using a microplate reader (Bio-Rad, Hercules, CA, USA).

### Protein assay

Protein levels were measured in the cell lysate supernatants in all samples using BCA kit (Pierce, Rockford, IL). Protein standards were obtained by diluting a stock solution of BSA. Linear regression was used to determine the actual protein concentration of each sample.

### Proinflammatory cytokine assay

After treatment period, supernatants were removed and stored at -70°C. Pro-inflammatory cytokine (IL-8 and IL-6) levels were measured using an ELISA employing a biotin-streptavidin-peroxidase detection system with the respective duo antibody kits (R&D Systems) according to the manufacturer's instructions. Each sample was assayed in triplicate and the values were expressed as mean of three experiments.

### Immunocytochemical analysis of NF-κB RelA/p65 localization

Activation of NF-κB in SAEC was assessed by immunocytochemical localization of RelA/p65 subunit of NF-κB. SAEC were seeded at 5000 cells/well in 8-well glass chamber slides and cultured overnight in SAGM at 37°C. Cells were then treated with CSE (1.0%) and TNF-α (10 ng/ml) as a positive control for 20 min. At the end of incubation, the cells were washed twice in PBS and fixed in 4% paraformaldehyde for 10 min at room temperature. The cells were permeabilized with 0.1% Triton X-100. The wash step was repeated and the cells were blocked with 10% normal goat serum for 1 hr. The cells were then incubated overnight in humidified chamber at 4°C, with rabbit polyclonal antibodies directed against the RelA/p65 subunit of NF-κB (Santa Cruz Biotechnology, USA), diluted at 1:200 in 1% goat serum in PBS. Furthermore, the cells were washed with PBS and incubated with FITC-labeled anti-rabbit IgG diluted 1:200 in 1% goat serum for 1 hr at room temperature in dark. After rinsing with PBS, the coverslips were mounted onto the slides and viewed under fluorescence microscope. Nuclear translocation of RelA/p65 was interpreted as a positive result from the fluorescence obtained.

### Western blot analysis for NF-κB RelA/p65

Primary human SAEC were exposed to different concentrations of CSE (0.5 and 1.0%) for 1 hr. After treatment, the cells were washed with ice-cold PBS and resuspended in buffer A (10 mM HEPES, pH 7.9, 10 mM KCl, 0.1 mM EDTA, 0.1 mM EGTA, 1 mM DTT, and 0.5 mM PMSF). After 15 min of incubation, Nonidet P-40 was added and the samples were centrifuged to collect the supernatant containing cytosolic proteins. The pelleted nuclei were resuspended in buffer B (20 mM HEPES, pH 7.9, 0.4 M NaCl, 1 mM EDTA, 1 mM EGTA, 1 mM DTT, and 1 mM PMSF) and kept on ice. After 30 min of incubation, the cell lysates were centrifuged, and supernatants containing the nuclear proteins were collected. Twenty μg of isolated nuclear protein from each group was analyzed by SDS-PAGE and transferred onto nitrocellulose membrane (Amersham, Arlington Heights, IL, USA) using electro-blotting technique. The nitrocellulose membrane was blocked with 10% nonfat dry milk for 1 hr at room temperature, and subsequently incubated with rabbit polyclonal NF-κB RelA/p65 (1:1000) in 5% nonfat dry milk overnight at 4°C. After three washing steps of 15 min each, NF-κB RelA/p65 protein levels were detected using goat anti-rabbit antibody (1:20,000) linked to horseradish peroxidase (Dako, Santa Barbara, CA, USA), and bound complexes were detected using an enhanced chemiluminescence method.

### Statistical analysis

Statistical analysis of significance was calculated using one-way Analysis of Variance (ANOVA) followed by Tukey's *post-hoc *test for multigroup comparisons using STATVIEW and Sigma plot statistical packages. The results were presented as the mean ± SEM of three independent experiments. *p < 0.05, ^#/^**p < 0.01, and ^§/^***p < 0.001.

## Results

### Cigarette smoke extract differentially induced cytotoxicity and reduced cell viability in a variety of alveolar epithelial cells and in primary human small airway epithelial cells

**Figure 1:**
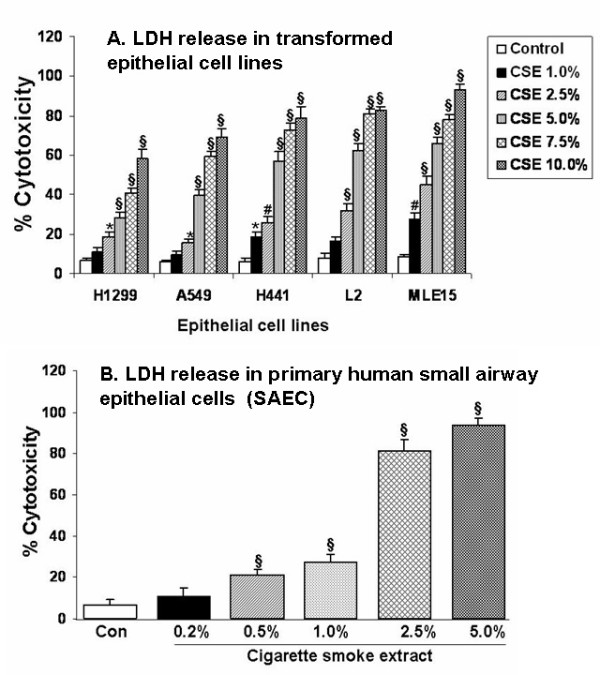
**Cigarette smoke extract differentially caused cytotoxicity in a variety of alveolar epithelial cells and in primary human small airway epithelial cells. A. **Various alveolar epithelial cells such as human lung cancer cells (H1299), human adenocarcinoma cells (A549), human lung epithelial cell from papillary adenocarcinoma patient (H441), rat lung epithelial cells (L2), and murine type II epithelial cells (MLE-15) were exposed to different concentrations of cigarette smoke (1R3F) extract (1.0–10.0%) for 24 hr, and % cytotoxicity induced was measured as lactate dehydrogenase release. CSE differentially induced cytotoxicity in concentration dependent manner in all the five epithelial cell lines. Amongst the five cell lines studied, H1299 cells were most resistant and MLE 15 cells were the least resistant. **B. **Primary human small airway epithelial cells (SAEC) were exposed to different concentrations of cigarette smoke (1R3F) extract (0.2–5.0%) for 24 hr and percentage (%) cytotoxicity induced was measured as LDH release. CSE dose-dependently induced LDH release in SAEC. Data represent mean ± SEM of 3 experiments. *p < 0.05, ^#^p < 0.01, and ^§^p < 0.001 compared to control group. CSE: cigarette smoke extract.

CSE differentially induced cell death in a concentration-dependent manner in various epithelial cell lines measured by LDH release assay (Figure 1A) and trypan blue exclusion assays (% cell viability at 5.0% CSE in H1299: 70 ± 3.9%; A549: 61 ± 5.4%; H441: 39 ± 2.1%; L2: 30 ± 1.4%, and MLE-15: 17 ± 2.7% versus control 100%, n = 3, p < 0.001). Among the cell lines studied, murine epithelial cells (MLE-15) were most sensitive to CSE followed by rat lung epithelial cells (L2). Among the human lung epithelial cells, H441 were most sensitive when compared with H1299 and A549. Furthermore, our results revealed that H1299 cells were most resistant among the five cell lines studied. On the whole, the sensitivity to CSE was in the order MLE15 > L2 > H441 > A549 > H1299. In case of SAEC, CSE dose-dependently induced cytotoxicity as assayed by LDH release (Figure 1B) and trypan blue exclusion assay (% cell viability at 0.2% CSE: 91 ± 3.2%; 0.5 % CSE: 85 ± 4.2%; 1.0 % CSE: 70 ± 3.5; 2.5 % CSE: 30 ± 2.1 and 5% CSE: 11 ± 2.5 versus control 100%, n = 3, p < 0.001). Cigarette smoke extract at concentrations above 1.0% was cytotoxic to SAEC.

### Cigarette smoke extract dose-dependently induced necrosis but not apoptosis in alveolar epithelial cells as well as in primary human small airway epithelial cells

**Figure 2:**
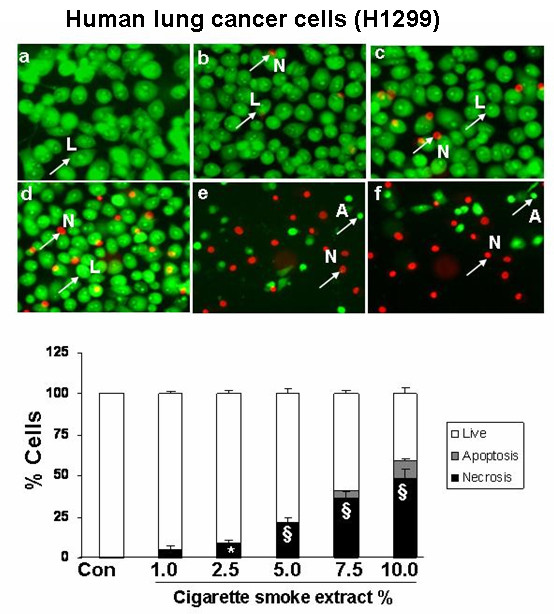
**Cigarette smoke extract caused necrosis with no or little evidence of apoptosis in human lung cancer cells (H1299)**. Human lung cancer cells (H1299) were treated with media alone (control) and various concentrations of CSE; a) control, b) CSE (1.0%), c) CSE (2.5%), d) CSE (5.0%), e) CSE (7.5%), f) CSE (10%) for 24 hr. The cells were stained with ethidium bromide and acridine orange and observed under fluorescence microscopy. Living cells had normal shaped nuclei with green chromatin. Early apoptotic cells have shrunken green nuclei with chromatin condensation, whereas necrotic or late apoptotic cells had normal/condensed nuclei that were brightly stained with ethidium bromide and appeared red. Percentage of viable (white bars), apoptotic (grey bars) and necrotic/late apoptotic (black bars) determined by counting as described in Materials and Methods. Results are mean of 3 experiments ± SEM. *p < 0.05, and ^§^p < 0.001 compared with control group. L = Live; A = Apoptosis; N = Necrosis.

**Figure 3:**
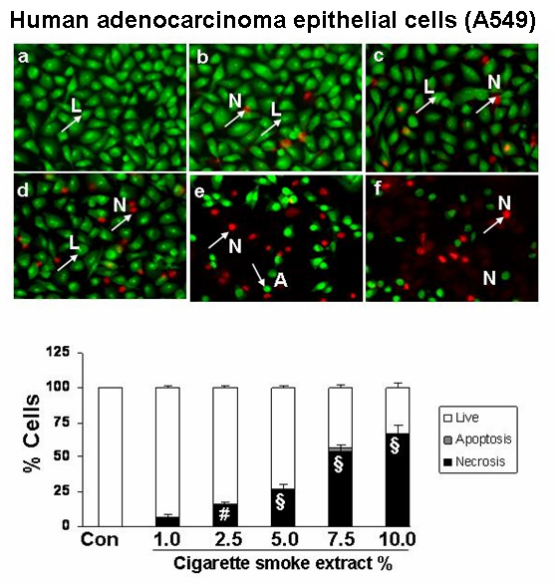
**Cigarette smoke extract induced necrosis with no or little evidence of apoptosis in human adenocarcinoma cells (A549)**. Human adenocarcinoma cells (A549) were treated with media alone (control) and various concentrations of CSE; a) control, b) CSE (1.0%), c) CSE (2.5%), d) CSE (5.0%), e) CSE (7.5%), f) CSE (10%) for 24 hr. Results are mean of 3 experiments ± SEM. ^#^p < 0.01, and ^§^p < 0.001 compared with control group. L = Live; A = Apoptosis; N = Necrosis.

**Figure 4:**
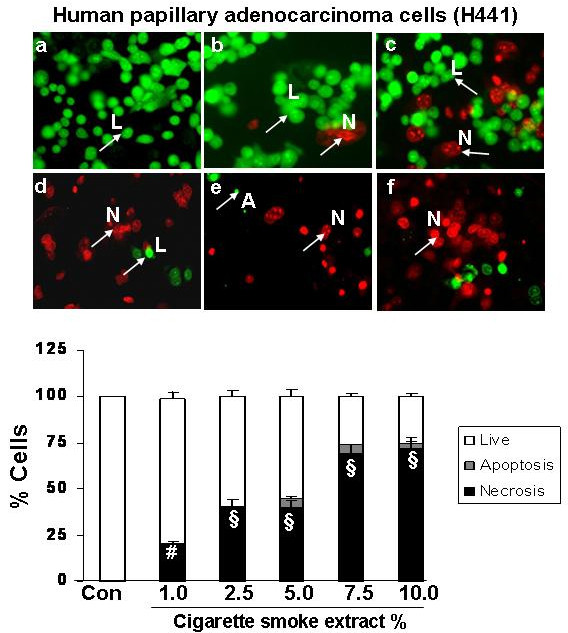
**Cigarette smoke extract caused necrosis with no or little evidence of apoptosis in human lung epithelial cell from papillary adenocarcinoma patient (H441)**. Human lung epithelial cell from papillary adenocarcinoma patient (H441) were treated with media alone (control) and various concentrations of CSE; a) control, b) CSE (1.0%), c) CSE (2.5%), d) CSE (5.0%), e) CSE (7.5%), f) CSE (10%) for 24 hr. Results are mean of 3 experiments ± SEM. ^#^p < 0.01, and ^§^p < 0.001 compared with control group. L = Live; A = Apoptosis; N = Necrosis.

**Figure 5:**
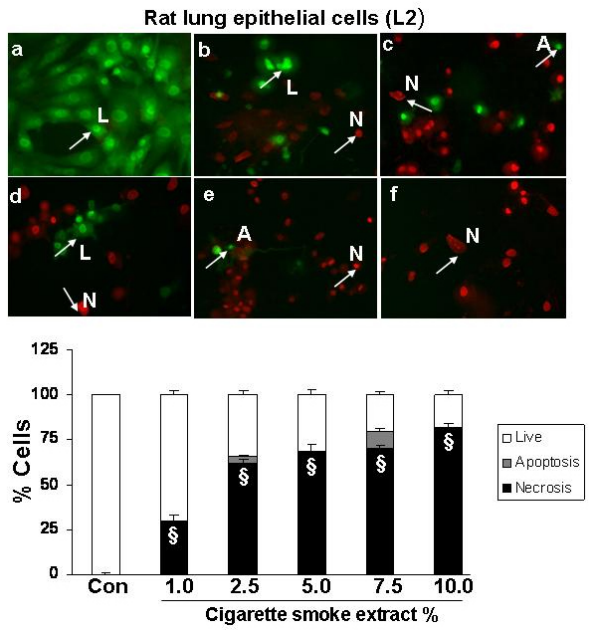
**Cigarette smoke extract caused necrosis with no or little evidence of apoptosis in rat lung epithelial cells (L2)**. Rat lung epithelial cells (L2) were treated with media alone (control) and various concentrations of CSE; a) control, b) CSE (1.0%), c) CSE (2.5%), d) CSE (5.0%), e) CSE (7.5%), f) CSE (10%) for 24 hr. Results are mean of 3 experiments ± SEM. ^§^p < 0.001 compared with control group. L = Live; A = Apoptosis; N = Necrosis.

**Figure 6:**
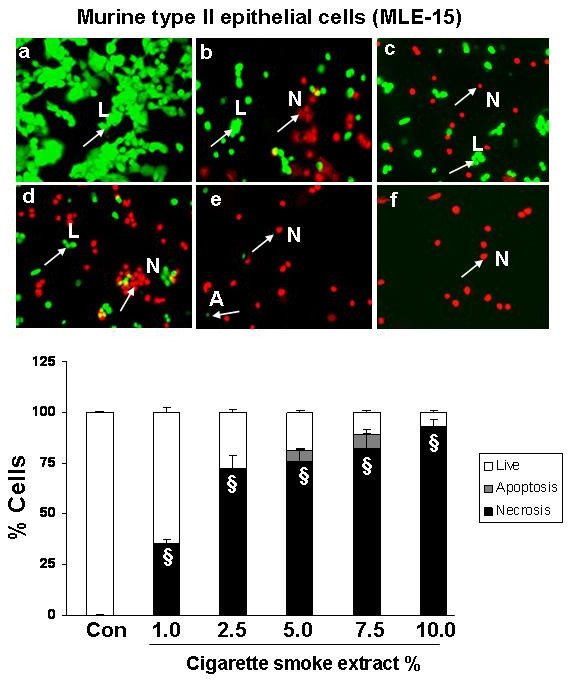
**Cigarette smoke extract caused necrosis with no or little evidence of apoptosis in murine type II epithelial cells (MLE-15)**. Murine type II epithelial cells (MLE-15) were treated with media alone (control) and various concentrations of CSE; a) control, b) CSE (1.0%), c) CSE (2.5%), d) CSE (5.0%), e) CSE (7.5%), f) CSE (10%) for 24 hr. Results are mean of 3 experiments ± SEM. ^§^p < 0.001 compared with control group L = Live; A = Apoptosis; N = Necrosis.

**Figure 7:**
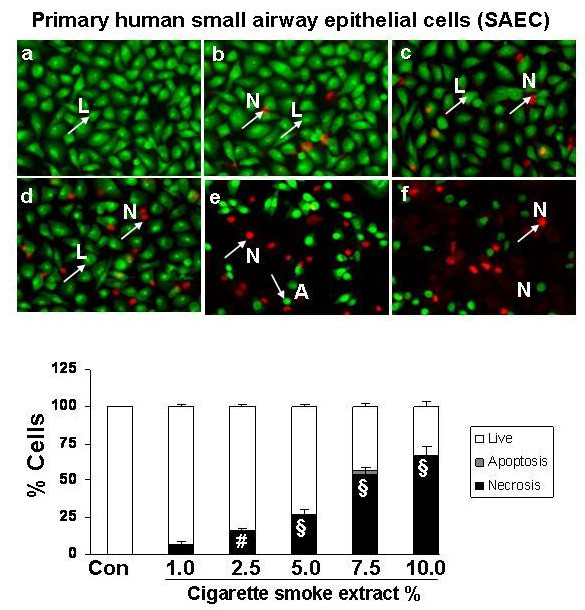
**Cigarette smoke extract caused necrosis with no or little evidence of apoptosis in primary human small airway epithelial cells (SAEC)**. Primary human small airway epithelial cells (SAEC) were treated with media alone (control) and various concentrations of CSE; a) control, b) CSE (0.2%), c) CSE (0.5%), d) CSE (1.0%), e) CSE (2.5%), f) CSE (5.0%) for 24 hr. Results are mean of 3 experiments ± SEM. ^#^p < 0.01, and ^§^p < 0.001 compared with control group. L = Live; A = Apoptosis; N = Necrosis.

To assess the degree of necrosis and apoptosis induced by CSE in various epithelial cell lines, the cells were double stained with acridine orange and ethidium bromide and the staining was observed under a fluorescent microscope. CSE induced necrosis in a dose- dependent manner in all the transformed epithelial cells as well as in human primary SAEC. The percentage of necrosis varied among the transformed epithelial cell lines at a given concentration of CSE. For example, necrosis caused by 5% CSE in various epithelial cell lines was as follows: H1299: 22 ± 3.6%; A549: 27 ± 1.5%; H441: 40 ± 5.8%; L2: 69 ± 4.3%; and MLE-15: 76 ± 5.2%; n = 3 (Figures 2, 3, 4, 5, 6, 7). CSE did not cause a significant degree of apoptosis in any of these epithelial cell lines.

### Cigarette smoke extract dose-dependently increased lipid peroxidation in alveolar epithelial cells and in primary human small airway epithelial cells

**Figure 8:**
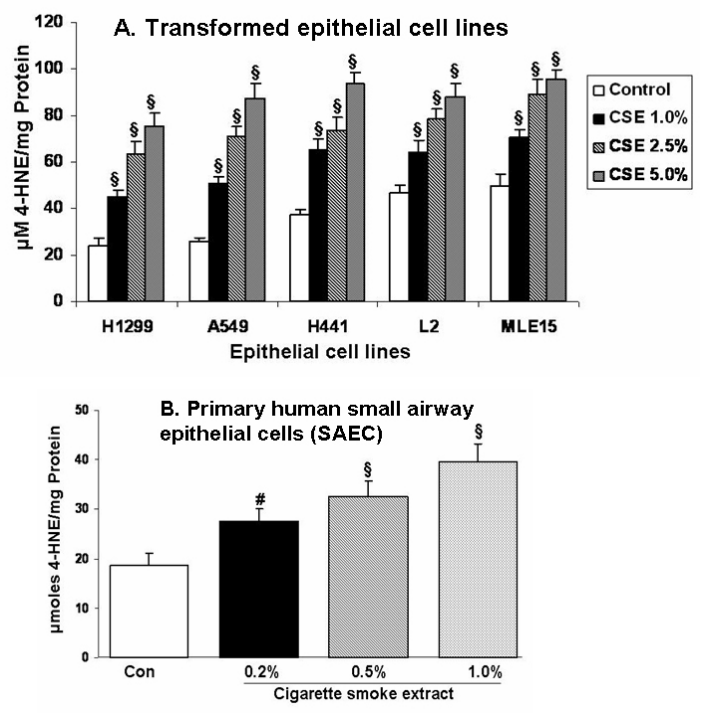
**Cigarette smoke extract dose-dependently caused lipid peroxidation measured as 4-hydroxy-2-nonenal levels in alveolar epithelial cells as well as in primary human small airway epithelial cells**. **A**. Transformed alveolar epithelial cells were exposed to cigarette smoke (1R3F) extract (1.0 – 5.0 %) for 24 hr and the extent of lipid peroxidation was determined by measuring 4-HNE levels. Cigarette smoke extract increased the levels of 4-HNE in all the five transformed alveolar epithelial cell lines in dose-dependent manner. However, the baseline levels of 4-HNE were varied amongst the cell lines, H1299 with lower base line levels and MLE-15 with higher baseline levels. **B. **Primary human small airway epithelial cells (SAEC) were exposed to cigarette smoke extract (0.2%-1.0 %) derived from 1R3F research grade cigarettes for 24 hr, and the levels of 4-HNE were measured. Cigarette smoke extract dose-dependently increased the levels of 4-HNE levels SAEC. Data represent mean ± SEM of 3 individual experiments. ^§^p < 0.001 compared to control values. CSE: cigarette smoke extract.

CSE dose-dependently increased the levels of 4-hydroxy-2-nonenal in all the five epithelial cell lines as well as in SAEC. However, the basal levels varied from one cell line to another, which were in the order of MLE15 > L2 > H441 > A549 > H1299 > SAEC. The levels of 4-hydroxy-2-nonenal levels correlated with degree of cytotoxicity induced by CSE in these cell lines (Figures 8A and 8B).

### Cigarette smoke extract decreased intracellular glutathione levels in various alveolar epithelial cells as well as in primary human small airway epithelial cells

**Figure 9:**
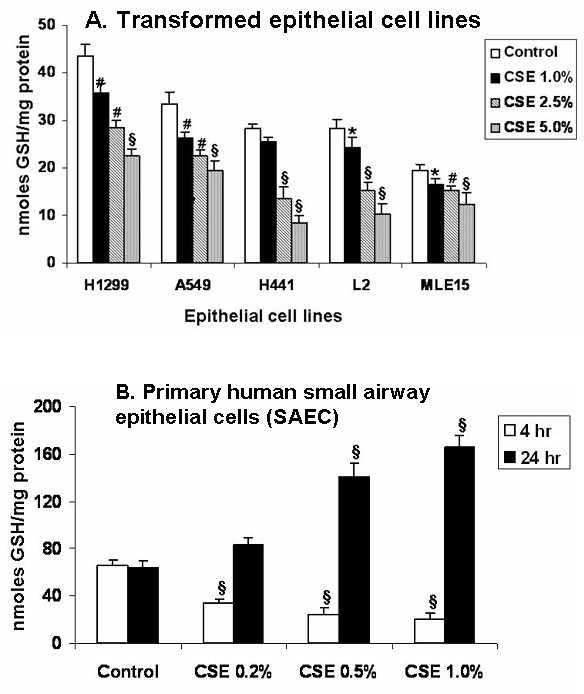
**Cigarette smoke extract showed differential effects on intracellular reduced glutathione levels in alveolar epithelial cells and in primary human small airway epithelial cells**. **A. **Transformed alveolar epithelial cell lines of our interest; H1299, A549, H441, L2 and MLE-15 were treated with cigarette smoke extract (1.0–5.0%) for 24 hr. After incubation period, GSH levels were measured by the Tietze method. Although, the baseline GSH levels were varied amongst the cell lines, CSE decreased GSH levels dose-dependently at 24 hr in all five epithelial cell lines. The most resistant cell line H1299 had higher baseline GSH levels whereas the least resistant MLE-15 had lower baseline GSH levels. **B. **Primary human small airway epithelial cells (SAEC) were also treated with cigarette smoke extract (0.2–1.0%) for 4 and 24 hrs, and GSH levels were measured. CSE dose- dependently decreased GSH levels in SAEC at 4 hr, where as the levels were increased dose-dependently at 24 hr. Data is representative of 3 separate experiments ± SEM. *p < 0.05, ^#^p < 0.01, and ^§^p < 0.001 compared with corresponding control. CSE: cigarette smoke extract.

Glutathione is involved in various biological events including redox signaling in the lungs. CSE decreased the levels of GSH in all the five cell lines studied in a dose-dependent manner (Figure 9A). CSE mediated GSH depletion was not associated with increased glutathione disulfide (GSSG) levels in A549 cells [8]. Interestingly, the baseline levels of GSH were varied based on their sensitivity to CSE amongst the different cell lines studied. CSE dose-dependently decreased the levels of GSH in SAEC at 4 hr, whereas the levels were increased dose-dependently at 24 hr (Figure 9B).

### Differential effects of cigarette smoke extract on proinflammatory cytokine release in transformed epithelial cells and in primary human small airway epithelial cells

Previously, we have shown that CSE treatment had no effect on A549 cells in terms of release of pro-inflammatory cytokines (IL-8) in A549 cells [9]. In this study, we investigated the pro-inflammatory effect of CSE in a variety of human as well as rodent alveolar epithelial cells (H1299, H441, MLE-15 and L2 in addition to A549) by using various concentrations of CSE (1.0–10%), and TNF-α as a positive control (10 ng/ml). Treatment with CSE showed insignificant proinflammatory cytokine (IL-8 and IL-6) release at 24 hr. However, TNF-α (10 ng/ml) significantly increased pro-inflammatory cytokine (IL-8 and IL-6) release at 24 hr (Table 1). In order to study whether whole cigarette smoke or direct cigarette smoke exposure to cells can induce pro-inflammatory cytokine release, we exposed A549 cells to mainstream smoke (10 μg of total particulate matter, TPM/m^3^) using a Baumgartner-Jaeger CSM2082i cigarette smoking machine [21] (CH Technologies, Westwood, NJ, USA), for 1 hr and then incubated without exposure for further 3, 6 and 24 hr as direct cigarette smoke exposure for longer than a few hours is cytotoxic. Proinflammatory cytokine (IL-8 and IL-6) release was measured in various supernatants. IL-8 release was not observed in A549 cells in response to whole cigarette smoke exposure (3 hr: 527 ± 35; 6 hr: 519 ± 41; 24 hr: 471 ± 29 versus control 510 ± 31 pg/ml, n = 3). This suggested that transformed lung epithelial cells do not produce pro-inflammatory cytokines in response to either CSE or whole smoke direct exposure. Interestingly, CSE caused release of proinflammatory cytokines (IL-8 and IL-6) in SAEC (Table 2). CSE also induced IL-8 and IL-6 release from normal human bronchial epithelial cells (data not shown).

**Table 1:** Cigarette smoke extract did not cause proinflammatory cytokine (IL-8 and IL-6) release in transformed alveolar epithelial cells

**Cell line**	**Treatment**				
	
	**Control**	**CSE (1.0%)**	**CSE (2.5%)**	**CSE (5.0%)**	**TNF-α (10 ng/ml)**
	**Interleukin-8 (IL-8) pg/ml**				
Human lung cancer cells (H1299)	51.3 ± 3.2	53.7 ± 4.7	56.5 ± 7.4	45.1 ± 3.1	432 ± 59.1***
Human adenocarcinoma cells (A549)	623 ± 52.9	635 ± 52.4	620 ± 80.1	612 ± 76.3	1200 ± 100***
Human papillary adenocarcinoma cells (H441)	200 ± 27.2	210 ± 35.1	200 ± 58.4	198 ± 39.2	384 ± 28.1***
	**Interleukin-6 (IL-6) pg/ml**				
Rat lung epithelial cells (L2)	40.2 ± 4.8	43.1 ± 5.2	41.5 ± 7.2	38.8 ± 2.6	165 ± 14.5***
Murine type II epithelial cells (MLE-15)	35.6 ± 2.3	31.4 ± 5.5	38.1 ± 2.3	31.9 ± 3.6	74.5 ± 5.7***

Alveolar epithelial cell lines (H1299, A549, H441, L2 and MLE-15) were treated for 24 hr with cigarette smoke extract (1.0–5.0%) prepared from 1R3F research grade cigarettes and TNF-α was used as a positive control (10 ng/ml). Cells were harvested, and supernatants were collected for the measurement of IL-8 and IL-6 levels by sandwich ELISA. Cigarette smoke extract treatment did not show any significant change in IL-8 and IL-6 release in any of the transformed cell lines, whereas TNF-α treatment induced proinflammatory cytokine (IL-8 and IL-6) release. Data represent mean ± SEM of 3 individual experiments. ***p < 0.001 compared to control values. CSE: cigarette smoke extract.

**Table 2:** Cigarette smoke extract dose-dependently caused induction of proinflammatory cytokine (IL-8 and IL-6) release from primary human small airway epithelial cells

**Proinflammatory cytokine (pg/ml)**	**Treatment**				
	
	**Control**	**CSE (0.2%)**	**CSE (0.5%)**	**CSE (1.0%)**	**TNF-α (10 ng/ml)**
Interleukin-8 (IL-8)	56.2 ± 7.1	126 ± 40.6***	171 ± 21.8***	418 ± 52.3***	591 ± 76.2***
Interleukin-6 (IL-6)	87. 3 ± 7.2	187 ± 43.5***	275 ± 31.6***	476 ± 54.8***	623 ± 51.7***

Primary human small airway epithelial cells (SAEC) were treated for 24 hr with cigarette smoke extract (0.2–1.0%) prepared from 1R3F research grade cigarettes and TNF-α was used as a positive control (10 ng/ml). Cells were harvested, and supernatants were collected for the measurement of IL-8 and IL-6 levels by sandwich ELISA. CSE treatment dose-dependently induced the release of both IL-8 and IL-6 from human SAEC. Data represent mean ± SEM of 3 individual experiments. ***p < 0.001 compared to control values. CSE: cigarette smoke extract.

### Effect of cigarette smoke extract on NF-κB translocation in primary human small airway epithelial cells

**Figure 10:**
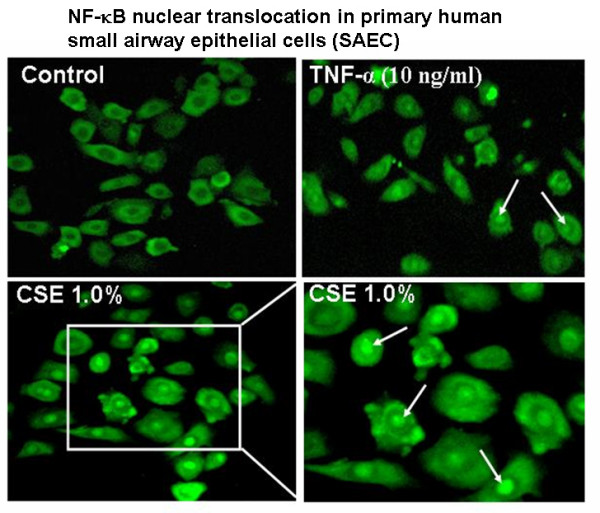
**Cigarette smoke extract treatment caused NF-κB RelA/p65 nuclear translocation in primary human small airway epithelial cells**. Primary human small airway epithelial cells were grown in 8-well chamber slides and were exposed for 20 min. to CSE (1.0%) prepared from 1R3F research grade cigarettes. TNF-α (10 ng/ml) was used as a positive. After treatment period, the cells were incubated with NF-κB RelA/p65 antibody and were visualized under fluorescent microscope. Cigarette smoke extract and TNF-α treatments caused nuclear translocation of NF-κB RelA/p65.

The expression of pro-inflammatory cytokines such as IL-8 and IL-6 are mediated by activation of the redox sensitive transcription factor, NF-κB. Previously, we have shown that CSE had no effect on activation of NF-kB in A549 cells [9]. In this study, we determined whether CSE can cause NF-kB translocation in SAEC since these cells showed a significant increase in proinflammatory cytokine (IL-8) release in response to CSE. Human SAEC showed significant degree of nuclear translocation of NF-κB in response to CSE at 20 min treatment (Figure 10).

### Cigarette smoke extract treatment increased NF-κB RelA/p65 levels in the nucleus of primary human small airway epithelial cells

**Figure 11:**
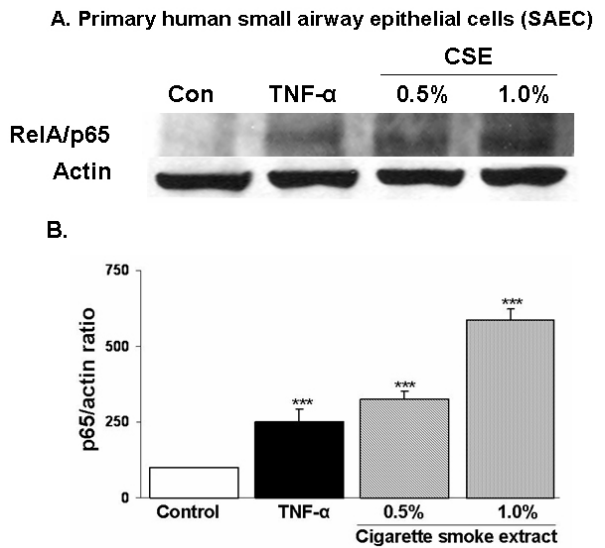
**Cigarette smoke extract mediated nuclear translocation of NF-κB RelA/p65 was associated with increased nuclear levels of NF-κB RelA/p65 protein in human primary small airway epithelial cells**. The primary human SAEC were treated with CSE (0.5 and 1.0%), and TNF-α (10 ng/ml) for 1 hr and nuclear proteins were isolated. Twenty microgram of nuclear protein was electrophoresed on SDS-PAGE and electroblotted onto membranes. A). Western blot showing increased nuclear levels of RelA/p65 in CSE and TNF-α treated SAEC at 1 hr. B). Nuclear protein levels of NF-κB p65 were expressed as the percentage of ratio of RelA/p65 versus actin in human SAEC. Each histogram is a representative of 3 separate experiments ± SEM. *p < 0.05, ^#^p < 0.01, and ^§^p < 0.001 compared with control. CSE: cigarette smoke extract.

In order to confirm CSE induced nuclear translocation of NF-κB in SAEC, nuclear proteins from SAEC treated with CSE were analyzed by western blotting. Cigarette smoke extract (CSE 0.5 and 1.0%) dose-dependently increased the levels of nuclear NF-κB RelA/p65 in SAEC after 1 hr treatment (Figures 11A and 11B).

## Discussion

Oxidative stress and inflammatory events, in response to cigarette smoke, play an important role in airway and alveolar epithelium injury [3,4,22]. In the present study, we investigated the effect of CSE on cytotoxicity, oxidative stress as well as pro-inflammatory cytokine release in a variety of alveolar epithelial cells (A549, H1299, H441, MLE-15 and L2), and compared the effect with primary human SAEC. CSE differentially induced cytotoxicity in various epithelial cell lines in a dose-dependent manner. Among the cell lines studied, rodent lung epithelial cell lines (MLE-15 and L2) were found to be more sensitive to CSE when compared to human lung epithelial cell lines (H1299, A549 and H441). This finding is supported by earlier studies showing that exposure of rat lung epithelial cells to lower concentrations of CSE resulted in a significant decrease in cell viability [7,9,23]. Our results also showed that human SAEC were highly sensitive to CSE compared with transformed epithelial cell lines. We postulate that the CSE induced cytotoxic effects may be due the presence of highly reactive electrophilic compounds (aldehydes and quinones) present in CSE [1,7,24], or the generation of intracellular ROS. Indeed, previous studies have shown that the cytotoxic ability of cigarette smoke [7,22,25-27] is due to a number of chemical components of cigarette smoke such as acrolein, nicotine, benzo(a)pyrene, and N- nitrosamines [28-30]. Nevertheless, our data clearly show that CSE caused toxicity to alveolar epithelial cells, which may contribute to the development of lung diseases induced by cigarette smoking [4,22,28].

Apoptosis is a well-defined programmed response that results in characteristic morphologic changes, such as cell shrinkage, condensation and fragmentation of nuclear material. Necrosis on the other hand, is a passive response characterized by cytoplasmic swelling, rapid loss of plasma membrane integrity, and eventually cell lysis [31]. We therefore studied the effect of CSE on cell death in various epithelial cells. Our data showed that CSE induced necrosis in a dose-dependent manner in all the five transformed epithelial cell lines as well as in SAEC. However, there was a significant variability in their sensitivity to different doses of CSE. Our data showing cigarette smoke induced necrosis with no or little evidence of apoptosis is in contrast to previous studies in macrophages and endothelial cells [26,32], where CSE was shown to induce apoptosis, but is in agreement with the earlier studies by Wickenden *et al *[17] who showed that cigarette smoke exposure inhibited apoptosis by preventing caspase activation, and instead promoted necrosis in alveolar epithelial (A549) cells. Furthermore, it is possible that the mode of cell death may be dependent on the cell type and the concentration of stimulus employed [33].

An imbalance between oxidants and antioxidants has been shown to occur in smokers [4,7,22] resulting in tissue injury. Such tissue damaging effects could be attributed to the presence of 10^14^–10^16 ^oxidantmolecules/puff of cigarette smoke [1]. 4-hydroxy-2- nonenal, a highly reactive and diffusible end product of lipid peroxidation, is a known marker of oxidative stress and can attack target cells far from the site of the original free radical event [34-36]. It is a potent alkylating agent, which reacts with DNA and proteins, generating various types of adducts [34,36,37] that are capable of inducing stress signaling pathways and apoptosis [37]. It is possible that 4-HNE is generated by CSE either directly or indirectly via lipid peroxidation of cell membranes. In this study, we have attempted to study whether any variations in the extent of lipid peroxidation induced by CSE in various epithelial cells is responsible for differential cytotoxicity. Indeed, our data showed that cigarette smoke extract dose-dependently caused increase in oxidative stress in all five cell lines and the baseline levels of 4-HNE were varied amongst the cell lines based on their sensitivity to CSE as observed for cytotoxicity. Furthermore, CSE dose-dependently increased the levels of 4-HNE in SAEC. This observation is corroborated with our previous findings that the levels of 4-HNE were increased in airways and alveolar epithelium of smokers and COPD subjects [38].

Glutathione (GSH) is a major intra- and extracellular antioxidant in the lung. We studied the effect of CSE on intracellular GSH levels in a variety of transformed epithelial cell lines and SAEC, since CSE has been shown to induce oxidative stress and alter glutathione homeostasis. Consistent to our hypothesis, we were able to show that treatment with CSE resulted in significant depletion of GSH levels in all the five epithelial cell lines without any significant change in GSSG levels (data not shown). It is possible that CSE mediated depletion of GSH levels could be due to the formation of GSH conjugates with electrophilic β-carbonyl compounds present in cigarette smoke as shown previously [8]. Moreover, our observation is consistent with earlier findings of the ability of cigarette smoke to induce oxidative stress by generation of reactive oxygen species and decrease intracellular GSH levels (without an increased levels of GSSG) in alveolar type II cells [8,20]. Interestingly, we observed differences in baseline GSH levels amongst the five epithelial cell lines studied. This potentially reflects the endogenous ability of the cells to adapt to cigarette smoke mediated injury and damage, which may in turn be attributed to the original intracellular concentration of GSH. However, this contention needs further experimentation. Another important finding was that CSE decreased GSH levels dose-dependently in SAEC at 4 hr time period. However, the levels were increased dose-dependently at 24 hr time period, which may be due to the rebound effect as a compensatory mechanism by up-regulation of glutamate cysteine ligase (glutathione biosynthesis) [39]. Overall, our findings on oxidant and antioxidant parameters suggest that CSE-induced cytotoxicity in different cell lines is due the base-line or endogenous levels of glutathione status and the amount of 4-HNE is formed.

IL-8 and IL-6 are important in the recruitment and activation of inflammatory cells. The induction of these pro-inflammatory mediators is regulated by the activation of redox sensitive transcription factor NF-κB [40,15]. This transcription factor has been shown to be activated by a wide variety of agents including stress, cigarette smoke, viruses, bacteria, inflammatory stimuli, cytokines and free radicals [40-43]. Previously we and others have shown that CSE caused activation of NF-κB and pro-inflammatory cytokines release in human monocytic cell line (MM6), Swiss 3T3, human histolytic lymphoma (U-937) and Jurkat T cells [13,44,45]. However, in another study by Moodie et al. CSE had no effect on either activation of NF-κB or pro-inflammatory cytokine release in A549 cells [9]. Hence, we investigated whether or not any other alveolar epithelial cell lines produce pro-inflammatory cytokines in response to CSE, which could potentially be used as a model to understand the mechanism of cigarette smoke-induced inflammatory events. We therefore, studied the effect of CSE on NF-κB activation in SAEC, and proinflammatory cytokine release in a variety of epithelial cell lines. Our data showed no effect of CSE on the release of pro-inflammatory mediators in any of the transformed alveolar epithelial cells studied. This observation is in agreement with our previous observation in A549 cells where CSE did not show activation of NF-κB and pro-inflammatory cytokine release [9]. Bihl *et al *have also showed that transformed human alveolar epithelial cells such as H1299 lack IL-6 production [46]. The reason for lack of pro-inflammatory effect of cigarette smoke in these cell lines is still unclear but it may be possible that the transformed epithelial cells have altered intracellular NF-κB and MAP kinase signaling mechanisms compared to normal cells. Interestingly, CSE induced both IL-8 and IL-6 release with a corresponding increase in nuclear translocation of RelA/p65 in SAEC as shown by immunofluorescent staining. Furthermore, western blot analysis of NF-κB RelA/p65 revealed increased levels of RelA/p65 in CSE treated SAEC cells compared with untreated SAEC. It is possible that apart from RelA/p65 (NF-κB), other transcription factors (such as AP-1, NF-IL6) may be responsible for CSE induced pro-inflammatory cytokine release. Our findings in SAEC gain credence from previous studies in human subjects wherein an increased expression of NF-κB was reported in the airway epithelium of smokers compared to nonsmokers [47]. Furthermore, increased levels of chemokines were also reported in alveolar type II cells obtained from smokers [48]. However, further investigations are required to understand the molecular signaling pathways involved in the pro-inflammatory effects of cigarette smoke in primary human airway epithelial cells

In conclusion, our data showed that CSE caused oxidative stress in a variety of alveolar epithelial cell lines as well as in primary human small airway epithelial cells. However, CSE triggered NF-κB activation and pro-inflammatory cytokine release in primary human small airway epithelial cells but not in any of the transformed epithelial cell lines studied. This study suggests that primary, but not transformed, lung epithelial cells are an appropriate model to study the inflammatory mechanisms in response to cigarette smoke in *in vitro *system.

## Abbreviations

CSE: Cigarette smoke extract

ELISA: Enzyme linked immunosorbent assay

FBS: Fetal bovine serum

GSH: Reduced glutathione

4-HNE: 4-Hydroxy-2-nonenal

IL-6: Interleukin-6

IL-8: Interleukin-8

LDH: Lactate dehydrogenase

NF-κB: Nuclear Factor *kappa *B

PBS: Phosphate buffered saline

SAEC: Small airway epithelial cells

TNF-α : Tumor necrosis factor-alpha

## Competing interests

The author(s) declare that they have no competing interests.

## Authors' contributions

AK performed all studies mentioned and drafted the manuscript. SY assisted with immunocytochemistry study. IR was involved in the design, supervision and writing of the manuscript. All authors read and approved the final manuscript.
