# Differential effects of cigarette smoke on oxidative stress and proinflammatory cytokine release in primary human airway epithelial cells and in a variety of transformed alveolar epithelial cells

**DOI:** 10.1186/1465-9921-9-6

**Published:** 2008-01-18

**Authors:** Aruna Kode, Se-Ran Yang, Irfan Rahman

**Affiliations:** 1 Department of Environmental Medicine, Lung Biology and Disease Program, University of Rochester Medical Center, Rochester, NY, USA

Since publication of our article [1], we have been made aware of several errors in our article.

In Figure 2 (Figure  1 in this paper) 'Cigarette smoke extract caused necrosis with no or little evidence of apoptosis in human lung cancer cells (H1299)' of our published article [1], panels d, e, and f show an identical red-staining pattern. The corrected figure, with the red staining pattern overlaying the green fluorescent staining of each group is given here as Figure 1.

**Figure 1:**
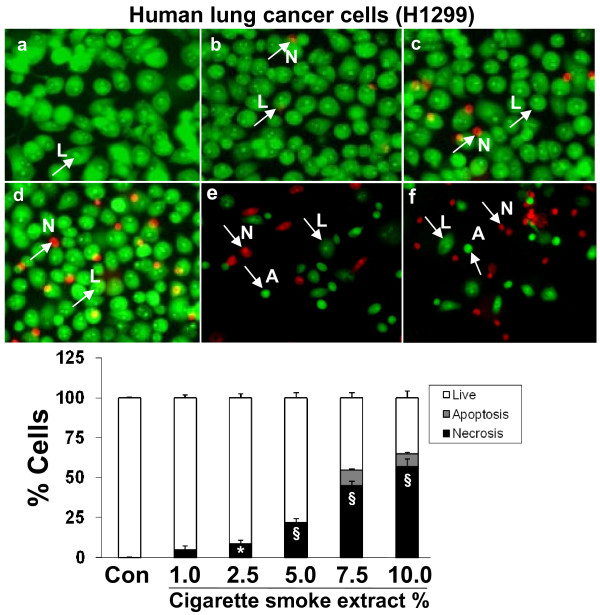
**Cigarette smoke extract caused necrosis with no or little evidence of apoptosis in human lung cancer cells (H1299)**. Human lung cancer cells (H1299) were treated with media alone (control) and various concentrations of CSE; a) control, b) CSE (1.0%), c) CSE (2.5%), d) CSE (5.0%), e) CSE (7.5%), f) CSE (10%) for 24 hr. The cells were stained with ethidium bromide and acridine orange and observed under fluorescence microscopy. Living cells had normal shaped nuclei with green chromatin. Early apoptotic cells have shrunken green nuclei with chromatin condensation, whereas necrotic or late apoptotic cells had normal/condensed nuclei that were brightly stained with ethidium bromide and appeared red. Percentage of viable (white bars), apoptotic (grey bars) and necrotic/late apoptotic (black bars) determined by counting as described in Materials and Methods. Results are mean of 3 experiments ± SEM. *p < 0.05, and ^§^p < 0.001 compared with control group. L = Live; A = Apoptosis; N = Necrosis.

**Figure 2:**
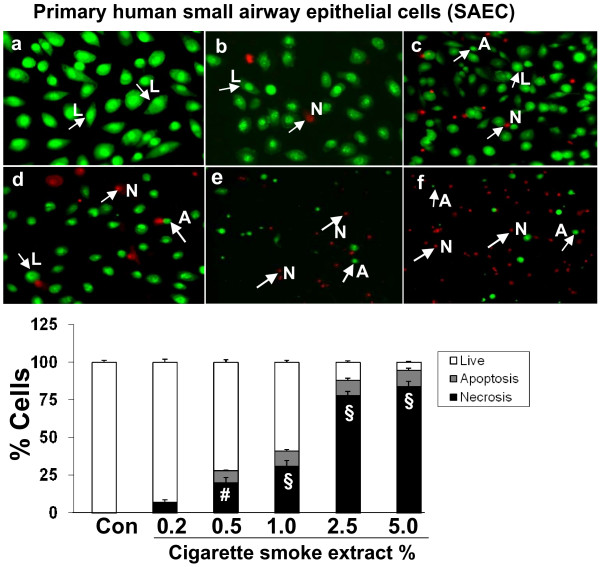
**Cigarette smoke extract caused necrosis with no or little evidence of apoptosis in primary human small airway epithelial cells (SAEC)**. Primary human small airway epithelial cells (SAEC) were treated with media alone (control) and various concentrations of CSE; a) control, b) CSE (0.2%), c) CSE (0.5%), d) CSE (1.0%), e) CSE (2.5%), f) CSE (5.0%) for 24 hr. CSE at higher concentrations was toxic to SAEC. Moreover, the cells underwent morphological changes in response to CSE and lost their characteristic spindle-shaped morphology as is evident from figs 2 c and d. SAEC were shown to be more sensitive to CSE compared to transformed cell lines. Results are mean of 3 experiments ± SEM. ^#^p < 0.01, and ^§^p < 0.001 compared with control group. L = Live; A = Apoptosis; N = Necrosis.

In addition in Figure 7 (Figure 2 in this paper) 'Cigarette smoke extract caused necrosis with no or little evidence of apoptosis in primary human small airway epithelial cells (SAEC)' of our published article [1], the cell morphology and histogram shown is identical to the cell morphology histogram given in Figure 3. The histogram for Figure 3 [1] is correct (and a corrected version of Figure 7 is given here as Figure 2) [1].

These errors inadvertently occurred during the preparation of these figures from the images of the fluorescent microscope. We sincerely apologize for the error and any inconvenience or confusion it may have caused.
